# Effect of red pepper (*Zanthoxylum bungeanum* Maxim.) leaf extract on volatile flavor compounds of salted silver carp

**DOI:** 10.1002/fsn3.1380

**Published:** 2020-02-03

**Authors:** Junke Li, Qiyi Liu, Jing Wang, Quanwen Liu, Zengqi Peng

**Affiliations:** ^1^ College of Food Engineering LuDong University Yantai China; ^2^ College of Food Science and Technology National Center of Meat Quality and Safety Control Nanjing Agriculture University Nanjing China

**Keywords:** dorsal muscle, lipid oxidation, principal component analysis, ventral muscle, volatile compounds

## Abstract

Chinese red pepper (*Zanthoxylum bungeanum* Maxim.) leaf (ZML) extract was added to salted silver carp. The effect of ZML extract on volatile compounds formation of both dorsal and ventral muscle of salted fish was investigated. Lipid oxidation of salted fish with ZML extract was alleviated with lower peroxide value (PV) and thiobarbituric acid reactive substance (TBARS) values than that of the control. Therefore, the contents of some volatile compounds formed mainly by oxidation such as benzene, methylbenzene, 1‐octene‐3‐ol, hexanal, and methyl ketone, which attributed off‐odor of salted fish, were reduced. Principal component analysis results showed that the first principal component (PC1) and the second principal component (PC2) explained 62% and 31% of total variance, respectively, and volatile compounds of the dorsal and ventral of control group differentiated from treatment group. These results showed that ZML extract can be a source of natural antioxidants and food additives for improving flavor of salted fish.

## Graphical abstract

Chinese red pepper (*Zanthoxylum bungeanum* Maxim.) leaf (ZML) extract was added to salted silver carp, the effect of ZML extract on volatile compounds formation of both dorsal and ventral muscle of salted fish were investigated. The results showed that ZML extract can be a source of natural antioxidants and food additives for improving flavor of salted fish.

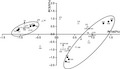

## 1 INTRODUCTION

Salting, as one of the techniques for preserving, has been applied to fish for long periods of time. Salted fish, are widely consumed mostly in coastal region of China, and the world at large for its unique flavor. Silver carp (*Hypophthalmichthys* molitrix), a commonly raised freshwater fish in China that may be the cheapest and most abundant fish, was selected as raw material to produce salted fish. However, the popularity of salted silver carp may be affected by the off‐odor which mainly was caused by earthy taste and lipid oxidation. In general, flavor (Fu, Lin, Xu, & Wang, 2015) and lipid oxidation (Mariutti & Bragagnolo, 2017; Salminen, Helgason, Kristinsson, Kristbergsson, & Weiss, 2017; Tan, Reyes‐Suarez, Indrasena, & Kralovec, 2017) of fish are researched as an entire fillet, dorsal, and ventral muscle have rarely been discussed, respectively. In fact, due to the differences in lipid content of dorsal and ventral muscles in fish (Testi, Bonaldo, Gatta, & Badiani, 2006), lipid oxidation of ventral muscle in salted sliver carp is greater than that of dorsal muscle in our earlier study (Li, Hui, et al., 2015); however, there is little information about differences in flavor between dorsal and ventral muscles in fish.

Some natural plants extracts or polyphenols have been reported by many researches (Jridi et al., 2017; Pahila, Kaneda, Nagasaka, Koyama, & Ohshima, 2017; Turgut, Işıkçı, & Soyer, 2017) could retard lipid oxidation of meat, while the effect of natural plants extracts or polyphenols on flavor of meat is rarely reported. Rivas‐Cañedo et al. (2013) investigated the supplement of Vitamin E reduced levels of lipo‐oxidation compounds mainly by lowering 2‐heptanone and 1‐penten‐3‐ol content. Bayberry polyphenols, showed the inhibition of lipid oxidation of yellowfin tuna fillets during refrigerated storage, could also reduce the sensory side‐effects (Bu et al., 2017). Red pepper (*Zanthoxylum bungeanum* Maxim.), as a traditional Chinese peppery spice, is used very normal in every family, but the leaf of the red pepper have not been taken full advantage. In our earlier study (Li, Hui, et al., 2015), we have identified the pepper leaf (ZML) of polyphenol compounds, chlorogenic acid, hyperoside, and quercitrin as the maximum content polyphenols, which have a good potential as an antioxidant for salted fish.

The aim of this study was to investigate the effect of ZML extract on the flavor of salted fish both in dorsal and ventral muscle and its sensory attributes.

## 2 MATERIALS AND METHODS

### 2.1 Preparation of ZML extract

ZML samples were collected from Taiyuan, Shanxi Province, China, in August 2015. The leaves were processed such as cleaned, dried, extracted, purified by macroreticular resin, and freeze‐dried following the method of Li, Hui, et al. (2015). The obtained ZML polyphenols was stored at 4℃.

### 2.2 Preparation of fish samples

Silver carp with the average weight of 1.5 ± 0.5 kg was obtained from a local farmers' market in Nanjing in September 2015. The fish was split by the method of Li, Hui, et al. (2015). The three fish were randomly sampled as control of the raw material; the remaining fish were salted with 4% salt. Total phenolic content of ZML extract was 555 mg/g, and so the addition of ZML extract added to salted fish was selected 0.03% (w/w). Then, ZML extract was added (w/w) by the method of Li, Hui, et al. (2015) following formulation: (a) control (no antioxidant added); (b) 0.030% ZML ethanol extract. For these two groups, the samples were salted with 4% salt for 2 days at 4℃ and 85%–90% relative humidity (RH) in plastic containers. During the process of drying, the temperature was increased to 17℃ and the RH was set to 85% on the 3rd day and 4th day, then the temperature was increased to 19℃ and the RH was set to 82% on the 5th day and 6th day, at last the temperature was increased to 21℃ and the RH was set to 79% on the 7th day and 8th day. The dorsal and ventral muscle were trimmed from the fish at the 4 process points (end of salting, drying‐processing 2, 4, and 6 days) and then was used to analyze. Samples were cut into small pieces, then vacuum‐packaged and stored at −20℃ until analysis. All of the analyses were performed in triplicate.

### 2.3 Chemicals

All of the chemicals used in the experiment were analytical grade. The standard substances (98%) was purchased from Zelang Biological Company. The other chemicals required for all biochemical assays were obtained from Sigma Chemical Co.

### 2.4 Total phenolic content

The total phenolic content of ZML extract was measured by the Folin‐Ciocalteu's phenol method of Li, Hui, et al. (2015). About 0.05 g of ZML extract powder was dissolved by 2 ml ethanol, the to 2 ml of Folin‐Ciocalteu's phenol reagent added, mixed, and left to stand for 3 min, then standing at ambient temperature for 1 hr after Adding 2 ml of a 10% (w/v) aqueous sodium carbonate solution. The absorbance was measured at 640 nm. The total phenolic content in ZML extract was determined using a standard curve prepared with chlorogenic acid and expressed as mg chlorogenic acid equivalent/ g leaf on a dry basis.

The total phenolic content of the ZML extract was 555 mg/g.

### 2.5 Lipid oxidation

The peroxide value (PV) and the thiobarbituric acid reactive substances (TBARS) assay were performed as described by Li, Wang, Li, and Peng, (2015). The PV values were expressed as m mol/kg muscle, and the TBARS values were expressed as mg malonaldehyde (MDA)/kg dry muscle.

### 2.6 Volatile compounds in salted fish by SPME/GC‐MS

The volatile compounds in salted fish were sampled by SPME/GC–MS. About 5.0 g sample was weighed and pulverized in 10 ml headspace vial, then immediately sealed and heated under water bath temperature of 80℃ for 35 min. After that, extraction fiber was inserted and the probe was placed on the sample. Taking probe from headspace vial after 2 min, then directly adopted thermal desorption with GC‐MS. A headspace sampler (75 µmCar/PDMS) and an A DB‐WAX column (30 m × 0.25 mm × 0.25 μm) (Supelco) were used for chromatographic separations. Thelium as carrier gas in operating conditions for GC at rate of 0.8 ml/min. The temperature program employed was as follows: 4 min at 40°C, raised to 70°C at 6°C/min, then a ramp of 10°C/min raised to 230°C, held for 7 min. The injector temperature was at 250°C throughout the analysis. Total run time was 25 min.

Then compared the sample mass spectral data to that of MANILIB, NSITDEMO, REPLIB, WLILEY four standard spectrum library for component identification. That the similar index (SI) and reverse similar index (RSI) were both greater than 800 could be regarded as a foundation of qualitative analysis. And peak area was used to quantify flavor substances.

### 2.7 Sensory evaluation

Salted fish were evaluated for its umami, tenderness, color, flavor, and overall accept ability by 20 numbers of the sensory panel who were required to be familiar with food sensory evaluation but had no specific training relevant to these products. Panelists were 20–40 years old, including five males and five females, five undergraduates, three postgraduate students, and two teachers. Before the panel members evaluated the products, verbal instructions were given to them. A five‐point scale was used by a modification of the method of Azad Shah, Tokunaga, Kurihara, and Takahashi (2009). Processed salted fish were steamed by an electric saucepan for 8 min before sensory evaluation, and coded samples were served at room temperature under bright light in separate booths. The panel members were asked to evaluate the umami, tenderness, color, flavor, and overall accept ability on a five‐point structured scale (1 means less umami, extremely tough or too salty, less attractive color, less flavor, and extremely dislike, respectively; 5 means most attractive umami, extremely tender, most attractive color, most intense flavor, and like extremely, respectively).

### 2.8 Statistical analysis

Statistical analyses were performed using the SPSS version 16.0 statistical package for windows (SPSS Inc.). One‐way analysis of variance (ANOVA) and correlation analyses were performed with SAS 8.2 (SAS Institute Inc.). The means were compared by Fisher's least significant difference (LSD) test. The level of significance was set at *p* < .05. Multivariate data analysis (PCA) was applied to obtain a more comprehensible overview of volatile compounds and to investigate possible correlations among salted fish.

## 3 RESULTS AND DISCUSSION

### 3.1 Changes of volatile compounds in control group during processing

#### 3.1.1 Changes of volatile compounds in dorsal muscle of control group during processing

The volatile compounds in cured fish products mostly were determined by decomposition and oxidation of lipid（Table 1）. There were 25 kinds of volatile compounds in final products, dorsal muscle of control group, including 1 alkane, 3 aldehydes, 9 alcohols, 2 ketones, 1 ether, 5 acids, 1 benzene hydrocarbons, 2 esters, and 1 oxime.

**Table 1:** Concentrations of volatile compounds both in dorsal and ventral of salted fish (relative content, %)

	Appearance time of peak (min)	Compounds	CD	CV	TD	TV
		Alkanes				
V1	20.3	Heptadecane	1.12 ± 0.04 ^c^	1.18 ± 0.06 ^c^	6.17 ± 0.15 ^b^	6.40 ± 0.14 ^a^
V2	4.13	Methylene Chloride	–	–	0.21 ± 0.01 ^b^	1.03 ± 0.10 ^a^
V3	2.6	Diazene, dimethyl‐	–	–	2.52 ± 0.38 ^b^	3.46 ± 0.45 ^a^
V4	17.6	Hexadecane	–	–	0.35 ± 0.04 ^a^	0.40 ± 0.07 ^a^
		Olefin				
V5	13	Styrene	–	–	0.27 ± 0.02 ^a^	0.28 ± 0.05 ^a^
		Aldehydes				
V6	7.93	Hexanal	5.91 ± 0.56 ^a^	5.46 ± 0.38 ^ab^	3.61 ± 0.16 ^c^	5.02 ± 0.39 ^b^
V7	2.61	Pentanal, 2,3‐dimethyl‐	–	4.35 ± 0.14 ^a^	–	–
V8	10.7	Heptanal	0.57 ± 0.09 ^a^	0.39 ± 0.05 ^b^	0.32 ± 0.05 ^b^	–
V9	17.8	Benzaldehyde	0.30 ± 0.05 ^b^	–	0.53 ± 0.04 ^a^	0.35 ± 0.06 ^b^
		Alcohols				
V10	4.34	Ethanol	2.86 ± 0.28 ^a^	–	1.86 ± 0.05 ^b^	–
V11	15.3	1‐Hexanol	1.65 ± 0.21 ^a^	1.17 ± 0.07 ^b^	0.62 ± 0.05 ^c^	0.36 ± 0.03 ^d^
V12	8.74	1‐Propanol, 2‐methyl‐	0.30 ± 0.04 ^c^	0.21 ± 0.04 ^c^	2.71 ± 0.19 ^a^	2.32 ± 0.10 ^b^
V13	10.9	1‐Penten−3‐ol	1.32 ± 0.04 ^b^	3.86 ± 0.25 ^a^	0.85 ± 0.10 ^c^	1.31 ± 0.07 ^b^
V14	13.3	1‐Pentanol	2.04 ± 0.09 ^a^	–	1.63 ± 0.07 ^b^	1.46 ± 0.04 ^c^
V15	12.2	3‐methyl−1‐Butanol	5.77 ± 0.26 ^a^	2.50 ± 0.10 ^b^	–	–
V16	14.6	(Z)−2‐Penten−1‐ol	0.99 ± 0.06 ^a^	–	–	–
V17	16.8	1‐Octen−3‐ol	0.48 ± 0.03 ^a^	0.49 ± 0.04 ^a^	–	–
V18	17	1‐Heptanol	0.26 ± 0.01 ^a^	–	–	–
V19	10.7	1‐Butanol	–	–	0.37 ± 0.03 ^b^	0.60 ± 0.04 ^a^
		Ketones				
V20	5.19	2‐Pentanone	–	3.13 ± 0.09 ^a^	–	–
V21	14.1	3‐hydroxy−2‐Butanone	4.33 ± 0.12 ^a^	2.85 ± 0.18 ^bc^	3.02 ± 0.10 ^b^	2.73 ± 0.13 ^c^
V22	7.36	2,3‐Pentanedione	0.52 ± 0.06 ^b^	2.79 ± 0.84 ^a^	0.32 ± 0.02 ^c^	–
V23	18	1‐(2‐furanyl)‐Ethanone			0.30 ± 0.03 ^b^	0.45 ± 0.06 ^a^
		Ethers				
V24	10	2‐Propanol, 1‐methoxy‐	–	–	1.33 ± 0.13 ^b^	0.70 ± 0.10 ^a^
V25	16.2	2‐butoxy‐Ethanol	6.76 ± 0.60 ^b^	5.77 ± 0.98 ^c^	10.27 ± 1.21 ^a^	2.14 ± 0.29 ^d^
		Acid				
V26	3.26	Pentanoic acid	0.14 ± 0.03 ^a^	0.14 ± 0.02 ^a^	0.09 ± 0.03 ^b^	0.09 ± 0.01 ^b^
V27	19.4	Butanoic acid	3.03 ± 0.34 ^a^	1.22 ± 0.17 ^b^	–	–
V28	19.9	3‐methyl‐Butanoic acid	0.23 ± 0.02 ^b^	1.33 ± 0.36 ^a^		
V29	18.2	Propanoic acid	0.45 ± 0.06 ^b^	1.16 ± 0.17 ^a^	–	–
V30	22	Hexanoic acid	7.27 ± 0.66 ^a^	–	–	–
		Benzene				
V31	4.32	Benzene	1.33 ± 0.04 ^c^	1.75 ± 0.15 ^a^	0.48 ± 0.12 ^bc^	0.64 ± 0.06 ^ab^
V33	9.34	p‐Xylene	–	–	0.37 ± 0.03 ^b^	0.60 ± 0.04 ^a^
		Ester				
V32	6.69	Toluene	0.63 ± 0.16 ^c^	0.79 ± 0.14 ^c^	11.6 ± 1.06 ^a^	9.18 ± 1.77 ^b^
V34	27.2	Diethyl Phthalate	4.33 ± 0.12 ^a^	2.85 ± 0.18 ^bc^	3.02 ± 0.10 ^b^	2.73 ± 0.13 ^c^
		Oxime				
V35	21	methoxy‐phenyl‐ Oxime	0.52 ± 0.06 ^b^	2.79 ± 0.84 ^a^	4.32 ± 0.02 ^c^	5.12 ± 0.46 ^c^

Note

Results are expressed as means ± *SD* (*n* = 3). Values in each row having the same letter are not significantly different (*p* > .05).

Abbreviations: CD, dorsal muscle of control group; CV, ventral muscle of control group; TD, dorsal muscle of treatment group; TV, ventral muscle of treatment group.

Alkanes and acids relative content in dorsal muscle of control group tended to decrease firstly and then increased, while the alkanes relative content of final dorsal products (1.12%) had not significantly changed (*p* > .05). The relative content of aldehydes of dorsal muscle in final products (6.78%) was lower than that of raw material (17.86%). Alcohols, ketones, and ethers relative content increased after salting (on the 2nd day), decreased to the lowest point at 6th day and then increased.

Benzene hydrocarbons in dorsal muscle of raw material was not detected until the 6th day, the relative content reached 0.28%, and then rose to 1.33% in final products. In dorsal muscle of control group, only on the 6th day, we found 0.50% phenol. It was only in raw material that amines were detected, and the relative content was 0.18%. The relative content of esters was 1.53% before processed, and then decreased, reaching 4.99% in the final products, which was 3.46% higher than that of raw material. Oximes mainly refers to methoxy‐phenyl‐oxime, whose relative content of raw material in the dorsal muscle was 0.48%, and the final products were 0.52%.

#### 3.1.2 Changes of volatile compounds in ventral muscle of control group during processing

There were 21 kinds of volatile compounds in ventral muscle, compared with dorsal muscle, the ventral muscle lacked 4 alcohols and 1 acid, but 1 ketone added.

In ventral muscle of control group, the relative content of alkanes in the raw material was 5.51%, which decreased firstly and then increased during processing. The relative content of olefins, aldehydes, alcohols, ketones, acids, and ethers in ventral muscle tended to increase at first and then decreased. Differing from dorsal muscle, aldehydes relative content of ventral muscle in final products (10.20%) was higher than that of raw material (9.5%). It is because the ventral had higher lipid contents and secondary oxidation of hydrogen peroxide occurs, producing more aldehydes.

However, benzene hydrocarbons in ventral muscle of raw material was 0.88%, increasing to 1.75% in final products. Phenol in ventral muscle of control group was also detected only at the 6th day, whose relative content was 0.25%. Amine was only detected in raw material, whose relative content was 0.37%. Only detected on 6th day, furan in ventral muscle accounted for 0.57%. The relative content of oxime was 0.79% in ventral muscle of raw material and increased to 2.79% in final products.

### 3.2 Changes of volatile compounds in treatments added ZML phenolic extract during processing

#### 3.2.1 Changes of volatile compounds in dorsal muscle of treatments added ZML phenolic extract during processing

The dorsal muscle of treatment group final products totally had 25 kinds of typical aroma substances. Compared with control group, the kinds of alkanes and olefins owned mild smell increased while the kinds of alcohol and acid which contribute to earthy smell decreased.

The relative content of alkanes in dorsal muscle rose from 2.47% (raw material) to 12.89% (at the 2nd day) by adding ZML phenolic extract. Then, it decreased and reached 9.25% on the 8th day, which was 6.78% higher than that of raw material. Aldehydes relative content tended to decrease, reducing from 17.86% (raw material) to 4.46% of final products.

Alcohols and esters reduced from raw material to the 2nd day, then increased to the 4th day, at last decreased to the 8th day. The relative content of ethers, acids, benzene hydrocarbons, and furan firstly decreasing and then gradually increased in dorsal muscle of treatment group. Phenols was not detected in the treatment during processing. The relative content of oxime rose from 0.48% of the original to 4.32%.

#### 3.2.2 Changes of volatile compounds in ventral muscle of treatments added ZML phenolic extract during processing

There were 22 kinds of volatile compounds in ventral muscle of final products, the kinds of volatile flavor components in ventral muscle changed by adding ZML alcohol extract, 3 aldehydes, 1 aldehyde, 4 alcohols, and 4 acids vanished while 1 alkane, 1 olefin, 1 ether, 2 alcohols, and 1 benzene hydrocarbon added.

The relative content of alkanes, ketones, and esters in ventral muscle of control group increased at first and then decreased. Aldehydes, alcohols, and acids relative content in ventral muscle of treatment group fell from raw material to the 2nd day, and rose at the 6th day, finally decreased on the 8th day.

Phenol and amine compounds was not detected in treatment group ventral muscle during processing. Oxime relative content in ventral muscle raw material was 0.79% and did not find in the final product.

### 3.3 Lipid oxidation of salted sliver carp

Thermal oxidation is one of the greatest threats to unsaturated fatty acids in foods. To investigate the relation between flavor and lipid oxidative deterioration, violent components for flavor and peroxide value (PV), thiobarbituric acid reactive substance (TBARS) values for lipid oxidation were evaluated.

**Figure 1:**
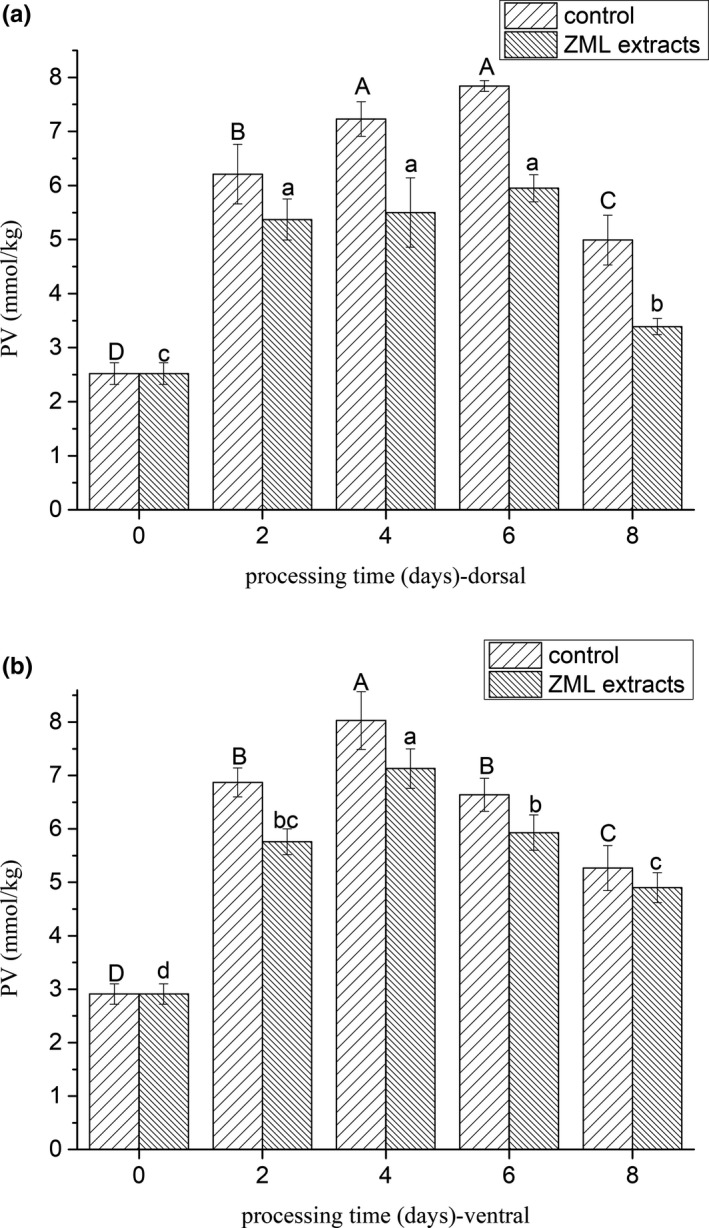
(a) Changes of PV in the dorsal muscle of salted fish. Note: Processing time 0–2 day means the salting period, 2–8 day means the drying period. (b) Changes of PV in the ventral muscle of salted fish. Note: Processing time 0–2 day means the salting period, 2–8 day means the drying period. Values in each column having the same capital letter are not significantly different of control group (*p* > .05); values in each column having the same lowercase capital letter are not significantly different of ZML extracts group (*p* > .05)

**Figure 2:**
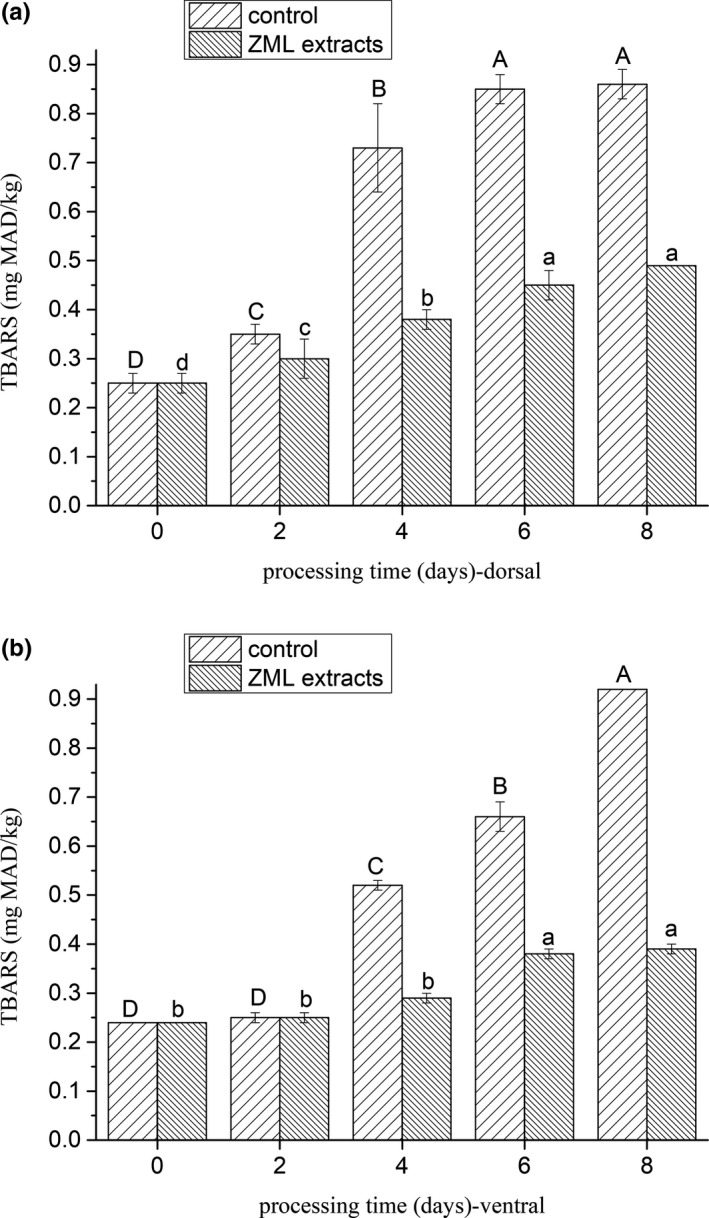
(a) Changes of TBARS in the dorsal muscle of salted fish. Note: Processing time 0–2 day means the salting period, 2–8 day means the drying period. (b) Changes of TBARS in the ventral muscle of salted fish. Note: Processing time 0–2 day means the salting period, 2–8 day means the drying period. Values in each column having the same capital letter are not significantly different of control group (*p* > .05); values in each column having the same lowercase capital letter are not significantly different of ZML extracts group (*p* > .05)

As shown in Figure 1, the primary lipid‐oxidation products, especially hydroperoxides was measured by PV (Simic & Taylor, 1987). PV of fresh fish dorsal muscle in control group was 2.52 mmol/kg muscle, it reached to 7.84 mmol/kg muscle at the 6th day, then decreased to 4.99 mmol/kg muscle at the 8th day. Compared with dorsal muscle, PV of ventral muscle reached to the highest value (8.03 mmol/kg muscle) at the 4th day and then decreased. The process temperature increased throughout the processing; therefore, the relative high temperature (19–21 ºC) could promote the PV to decrease significantly (*p* < .05) by leading the hydroperoxides to be further oxidized to secondary oxidation products (Chaijan, Benjakul, Visessanguan, & Faustman, 2006). The TBARS values were used to measure the lipid‐oxidation development in terms of secondary oxidation products. TBARS values both in the dorsal and ventral, whichever of the control and treatments, increased through the whole processing (Figure 2). This indicated that the lipids underwent oxidation during the drying period. The trend of TBARS values was corresponded with the decrease in the PV during the same period, the rate of hydroperoxide formation was equal to the rate of hydroperoxide decomposition into secondary oxidation products (Nguyen, Thorarinsdottir, Thorkelsson, Gudmundsdottir, & Arason, 2012).

ZML extract influenced PV and TBARS values of the treatment. Adding ZML extract resulted in a lower lipid oxidation of treatments, as indicated by the lower PV and TBARS values throughout the entire process period when compared with that of the control (*p* < .05). These results were in accordance with many recent studies (Leticia, Paola, Jordi, Julio, & Sancho, 2017; Zhang et al., 2017), showing that natural plant extract could decrease the lipid oxidation of meat.

Lipid oxidation, corresponding to the oxidative deterioration of polyunsaturated fatty acids in fish muscle, leads to the production of off‐flavor, thereby reducing the quality of food (Azad Shah et al., 2009). With respect to volatile compounds of salted fish, lipid oxidation might somewhat affect the formulation of volatile compounds. Such as benzene and toluene, as common flavor components of salted fish was generally generated by lipid oxidation and phenylalanine decomposition, would produce unpleasant flavor for fishes; 1‐octene‐3‐ol, one kind of alcohols, was produced by unsaturated fatty acid oxidation, endows the earthy smell to salted silver carp; hexanal, generally generating from linoleic acid oxidation, was regarded as the peroxidation reaction product of PUFA, could indicate the lipid oxidation together with conjugated diene value; methyl‐ketone, as typical products of lipid oxidation, there was 2‐butanone and 2‐pentanone in this study. ZML extract could effectively inhibit lipid oxidation of salted fish; thereby, the undesirable volatile compounds above could also be decreased, or even eliminated.

### 3.4 Effect of ZML phenolic extract on the flavor of salted sliver carp

According to the results, we could draw that the relative content of alkanes in dorsal and ventral muscles of control final products were respectively 1.12%, 1.18%, and turned to 9.25%, 11.29% after adding ZML alcohol extract. However, due to the relatively high threshold of alkanes compounds, which owned mild odor, could not generate the earthy smell.

Benzene hydrocarbons, such as benzene and toluene, generally generating from lipid oxidation and phenylalanine decomposition, were common volatile components of salted fish and would produce unpleasant flavor for fishes (Berdague, Denoyer, Le Quere, & Semon, 1991). The relative contents of benzene hydrocarbons in dorsal and ventral muscle of the control group final products were 1.33% and 1.75%, decreased to 0.48% and 0.64%, respectively, after adding ZML alcohol extract, which could reduce the unpleasant flavor of salted fish effectively.

Alcohols relative content was high, and correspondingly contributing more to the earthy smell of salted fish, for example 1‐octene‐3‐ol. The relative content of alcohols in dorsal and ventral muscle of the control were 15.67% and 8.23%, decreasing to 8.04% and 6.05%, respectively, in the treatment, greatly reduced the earthy smell. For example, 1‐octene ‐3‐ ol, produced by oxidation of unsaturated fatty acid. The relative content in dorsal and ventral muscle of control group was 0.48% and 0.49% and could not be detected in treatment group by adding ZML extract, the phenomenon could be illustrated that ZML alcohol extract can effectively reduce oxidation of unsaturated fatty acids. As for 1‐ pentene ‐ 3 ‐ ol, the relative content in dorsal and ventral muscle of control group was 1.32%, 3.86%, and reduced to 0.85% and 1.31% after handling with ZML alcohol extract, respectively were decreased by 0.47% and 2.55%, respectively.

The proportion of aldehydes was large, and also contributed to earthy smell, especially hexanal, nonanal, octanal, and other olefinic aldehydes (Zhou & Wang, 2006). Flavor of aldehydes was in accordance with the number of carbon atoms．Generally, aldehydes contained 3–4 carbon atoms produced strong pungent flavor while 5–9 carbon atoms of aldehydes emitted fragrance of lipid and oil. In addition, aldehydes with high molecular weight had unique fragrance of orange peel, such as the fresh grass smell of octanal and nonanoic acid, apple blossom fragrance of 2‐hexenal, pleasant sweet or fruity sweet taste of branched aldehydes, and cheese, nuts and salty taste of methyl aldehyde. The relative content of aldehydes in dorsal and ventral muscles of control group was 6.78% and 10.20%, and decreased to 4.46% and 5.37%, respectively, after adding ZML alcohol extract．The content of aldehydes reduced by 2.32% and 4.83%, respectively, having significant impact on reducing earthy smell of salted fish. Hexanal, as oxidation product of linoleic acid, which was regarded as the peroxidation reaction product of PUFA, together with conjugated diene value was considered as important indicators of lipid oxidation (Frankel, 1993; Frankel & Tappel, 1991). In the meantime, hexanal, a quite stable volatile component with relatively higher content, was main oxidation product and flavor compound in the dry‐cured meat products. Hexanal distributes corrupt taste of lipid in high concentration while it produces the smell of fresh grass and vegetables in low concentration. After adding ZML ethanol extract, the relative content of hexanal in dorsal decreased from 5.91% to 3.61%, dropping from 5.46% to 5.02% in the ventral. The reduction of hexanal relative content had crucial influence on preventing salted fish products from excessive oxidation and improving the flavor of salted fish．Benzaldehyde, as a kind of aromatic aldehydes, has pleasant fruity and nutty taste, which is an important volatile compound of products．The relative content of benzaldehyde in ventral muscle of control group was 0.30%, while not detected in ventral muscle．Dorsal and ventral muscle of treatment contained 0.53% and 0.35% benzaldehyde, respectively．Therefore, ZML alcohol extract can increase aromatic aldehydes content and produce pleasant fragrance.

Ketones relative content in final salted fish fell from 8.65% and 8.77% in dorsal and ventral muscle (the control) fell to 3.64% and 3.18% of treatment. Methyl‐ketone is a typical product of lipid oxidation (De Frutos, Sanz, & Martinez‐Castro, 1991), such as 2‐Butanone and 2‐Pentanone in this study. 2‐Pentanone was not detected in dorsal of control group while the content in ventral muscle was 3.13%．However, in the treatment group, 2‐Pentanone was not found in the ventral muscle.

Oxime is a organic compound produced by the reaction of aldehyde or ketone containing carbonyl and hydroxylamine．The trans‐oxime has sweet taste while cis‐oxime is tasteless, which has no harmful impact on the flavor of salted fish. Oxime in salted fish is methoxy‐phenyl‐oxime, whose relative content in dorsal and ventral muscle rose from 0.63%, 0.79% (control group) to 11.60%, 9.18% (treatment group) in final salted fish. It can be speculated that after adding ZML alcoholic extract, aldehydes and ketones, which contribute much to earthy smell of salted fish, partly reacted with hydroxylamine generating oxime.

Acetic acid may be obtained by the hydrolysis of triglycerides and phospholipids, whose relative content in dorsal and ventral muscle decreased from 2.40%, 2.14% (control group) to 1.44%, 1.71%, respectively．Acetic acid mainly neutralizes alkaline flavor compounds of salted fish such as amines. The formation process of esters is extremely complex, which was mainly formed by the esterification reaction involved with microorganisms. The relative content of esters both in dorsal and ventral muscle of salted fish were not high．Besides, due to the higher threshold than aldehyde, ester made no significant contribution to the total taste of salted fish.

From what have been discussed above, benzene/benzene derivatives, alcohols, aldehydes, and ketones contents both in dorsal and ventral were decreased by adding ZML extract. This result was supported by the recent work of Xi et al., who found that there was appositive correlation between benzene/benzene derivatives and alcohols with lipid oxidation in ready‐to‐eat turkey meat products.

### 3.5 Principal component analysis

A principal component analysis (PCA) was performed to establish which compounds were more valuable to salted fish treated with ZML alcoholic extract. The generated data set accounted for 93% of the total variance, where 62% and 31% of the variance were explained by PC1 and PC2, respectively.

**Figure 3:**
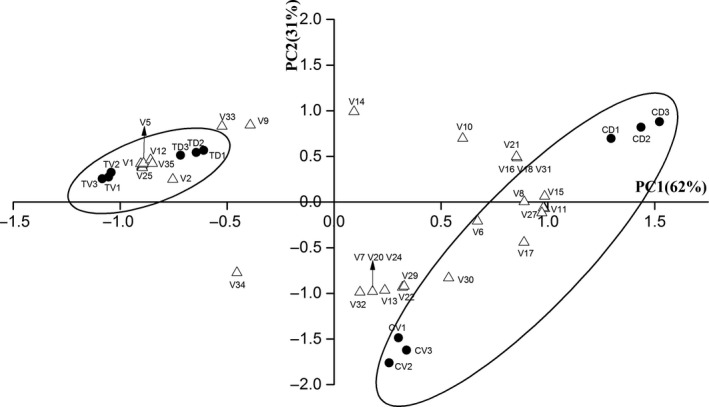
Principal component analysis of volatiles of salted fish with ZML extract (coefficients magnitude > 0.8) and locations. Note: V1‐V35 Numbers used in Figure 3 stand for the compounds in the same row in Table 1. CD—dorsal muscle of control group, CV—ventral muscle of control group, TD—dorsal muscle of treatment group, TV—ventral muscle of treatment group

The location of dorsal and ventral muscle of control and treatment group was described in Figure 3. Dorsal muscle of control group (CD) mainly in the first quadrant of the plot, contained higher amount of many volatile compounds, such as ethanol, 3‐hydroxy‐2‐butanone, 1‐heptanol, (Z)‐2‐penten‐1‐ol, benzene, and heptanal, while the ventral of control group (CV) in the fourth quadrant, mainly contained hexanal, butanoic acid, 3‐methyl‐butanoic acid, 1‐octen‐3‐ol, propanoic acid, hexanoic acid, 1‐penten‐3‐ol, and 2‐methyl‐3‐octanone. The lipid content of dorsal and ventral muscle differentiated volatile compounds of CD and CV group. Dorsal muscle (TD) and ventral muscle (TV) of treatment group were primarily in the second quadrant of the plot, evidencing a higher similarity among their aromatic qualities. The volatile compounds were dominated by heptadecane, 2‐butoxy‐ ethanol, 2‐hydroxy‐3‐pentanone, dimethyl‐diazene, styrene, 2‐methyl‐1‐propanol, didodecyl phthalate, methylene chloride, benzaldehyde, and p‐xylene.

The PCA result indicated that there was a significant (*p < .*05) difference between dorsal and ventral muscle of both control and treatment group. ZML extract, played a significant role in affecting and modulating the compositional profile due to its antioxidation and own flavor.

### 3.6 Sensory evaluation

To determine the effect of the ZML extract on the salted fish both in the dorsal and ventral muscle, the results obtained in the sensory analysis are presented in Table 2. From the results, we can see that the addition of ZML extract significantly improved the umami, tenderness, color, and flavor of salted fish both in the dorsal and ventral muscle compared with the control (*p* < .05). Especially the flavor of dorsal and ventral, the scores of treatments were 2.15‐ and 1.93‐fold compared with that of the control, respectively. The ZML extract in this study effectively improved overall acceptability score comparing with the control samples both of dorsal and ventral (*p* < .05), which coincides with the results of volatile flavor compounds of salted fish above. It is also meaningful to investigate the effect of ZML extract on flavor of salted fish in the dorsal and ventral muscle, respectively.

**Table 2:** Sensory evaluation of salted fish product with added ZML extract both in dorsal and ventral muscles

Antioxidant	Umami	Tenderness	Color	Flavor	Overall acceptability
Dorsal					
Control	3.18 ± 0.28^c^	3.32 ± 0.40^c^	2.42 ± 0.33^c^	1.98 ± 0.25^d^	2.46 ± 0.34^d^
ZML extract	4.22 ± 0.41^a^	4.17 ± 0.45^a^	3.58 ± 0.26^a^	4.26 ± 0.44^a^	4.35 ± 0.26^a^
Ventral					
Control	2.51 ± 0.35^d^	2.26 ± 0.27^d^	3.07 ± 0.14^c^	1.94 ± 0.27^d^	2.28 ± 0.26^d^
ZML extract	4.02 ± 0.25^b^	3.92 ± 0.25^b^	4.02 ± 0.11^a^	3.75 ± 0.28^b^	3.88 ± 0.20^b^

Note

Different letters within a column (a–d) are significantly different (*p* < .05).

There were certain correlations (Table 3) between overall acceptability of sensory evaluation and several kinds of typical volatile compounds relative contents both in dorsal and ventral muscle of salted fish. In conclusion, alcohols, hexanal, and methyl ketones relative content decreased, whereas the overall acceptability of sensory evaluation increased. And there was positive correlation between overall acceptability and benzaldehyde relative content. The treatment with ZML extract showed more significant correlation between overall acceptability and alcohols, hexanal, benzaldehyde, and methyl ketones relative contents in salted fish.

**Table 3:** Correlation between Overall acceptability of Sensory evaluation and several kinds of typical volatile compounds relative contents both in dorsal and ventral muscle of salted fish

Overall acceptability	Treatment	Correlation coefficient (*r*) & *p*‐value			
		Dorsal		Ventral	
Alcohols	Control	−0.998	<.01**	0.197	.71
	ZML extract	−1.000	<.01**	−0.999	<.01**
Hexanal	Control	−0.443	.52	−0.963	<.01**
	ZML extract	−0.806	.05	−0.923	<.01**
Benzaldehyde, 2,5‐bis[(trimethylsilyl)oxy]	Control	0.756	.08	‐	‐
	ZML extract	0.115	.83	0.904	.01*
Methyl ketones	Control	−0.791	.03*	−0.189	.72
	ZML extract	−0.933	<.01**	–0.792	.06
Benzene	Control	0.230	−.459	0.66	.36
	ZML extract	0.084	.471	0.87	.35

* *p* < .05.

** *p* < .01.

As can be seen from the results, the ZML extract played an important role in improving the flavor of salted fish and exhibited strong antioxidant activity by reducing oxidative products such as alcohols, hexanal, and methyl ketones.

## 4 CONCLUSIONS

ZML extract played an important role in the protection against lipid oxidation of salted fish therefore decreased or eliminated the undesirable volatile compounds generated during processing. There were totally 25 kinds of volatile compounds in dorsal muscle of control group, and 21 kinds in the ventral. Adding ZML extract had no effect on the total number of volatile components in dorsal muscle, but the kinds of alkanes and olefins owned mild smell increased while the kinds of alcohol and acid which contribute to earthy smell decreased. Compared with the dorsal, the kinds of volatile flavor components in ventral muscle changed by adding ZML alcohol extract, 3 aldehydes, 1 aldehyde, 4 alcohols, and 4 acids vanished while 1 alkane, 1 olefin, 1 ether, 2 alcohols, and 1 benzene hydrocarbon added. From the PCA result, volatile compounds of both dorsal and ventral muscle of salted fish added ZML extract were differenced from that of control group. The results suggest that ZML extract can be a source of natural antioxidants and food additives, and it is meaningful to investigate the flavor effect of ZML extract on salted fish both in dorsal and ventral muscle.

## CONFLICT OF INTEREST

The authors declare that they have no conflict of interest.

## ETHICAL APPROVAL

This study does not involve any human or animal testing.

## INFORMED CONSENT

Written informed consent was obtained from all study participants.
